# Cytotoxic and genotoxic action of Tagetes patula flower methanol extract and patuletin using the Allium test

**DOI:** 10.3906/biy-1906-7

**Published:** 2019-10-14

**Authors:** Mudassar AZHAR, Ahsana Dar FAROOQ, Sayedul HAQUE, Samina BANO, Lubna ZAHEER, Shaheen FAIZI

**Affiliations:** 1 Dr. Panjwani Center for Molecular Medicine and Drug Research, International Center for Chemical and Biological Sciences, Faculty of Toxicology, University of Karachi, Karachi Pakistan; 2 H.E.J. Research Institute of Chemistry, International Center for Chemical and Biological Sciences, Faculty of Pharmacology, University of Karachi, Karachi Pakistan; 3 Hamdard Al-Majeed College of Eastern Medicine, Faculty of Eastern Medicine, Hamdard University, Karachi Pakistan; 4 Department of Animal Sciences, Human Genetics Program, Quaid-i-Azam University, Islamabad Pakistan

**Keywords:** * Tagetes patula*, patuletin, *Allium*test, cytotoxic, genotoxic, chromosomal abnormalities

## Abstract

* Tagetes patula *is used to treat cancer patients in alternative healthcare systems. However, its cytotoxic and genotoxic effects have not been reported. Therefore, the**methanol extract of* T. patula *flower, the ethyl acetate fraction, and the pure compound patuletin were evaluated**using the *Allium* test.**The methanol extract and fraction contained ~3% and ~36% patuletin, respectively, with ~98% purity. The methanol extract caused inhibition of *Allium* root growth displaying an IC_50_ value of ~500 µg/mL, while the fraction and patuletin were more potent by ~2 and ~5 times, respectively. The *Allium* root tips demonstrated a decline in prophase, metaphase, anaphase, and telophase stages with concomitant decrease in percent mitotic index in the methanol extract (~5.64), fraction, and patuletin (~4) as compared to the control (~7.61). However, in only methanol extract-treated root tips, an increase in metaphase stage was noted. In addition, the methanol extract predominantly induced c-type, misaligned, and multipolar chromosomal abnormalities while the fraction and patuletin displayed fragments and sticky chromosomes. The fraction and patuletin also produced micronuclei (~2%)*. *In conclusion,* T. patula *flower methanol extract and ethyl acetate fraction are cytotoxic**and genotoxic, which most likely could be due to the patuletin. Further preclinical and clinical studies are required to justify its clinical use.

## 1. Introduction

*Tagetes patula* belongs to the family Asteraceae. Commonly known as French marigold, it is found abundantly in tropical regions and is also cultivated in Pakistan (Priyanka et al., 2013). *T. patula* has long been used in relieving the complaints of cough, dysentery, liver ailments, stomach ailments, and rheumatism (Vasilenko et al., 1990).****Commercially, it is used for the production of high-quality perfumes (Rajalakshmi and Jose, 2004). 

Pharmacologically, the**methanol extract (ME) of* T. patula* exhibited antiinflammatory activity (15 and 50 mg/mL) in mice due to lipoxygenase enzyme inhibition, which has been linked to its isolated flavonoidal compound, patuletin (Yasukawa and Kasahara, 2013). Its leaves are useful in relieving muscle- and kidney-associated pain (Rahman et al., 2013). The antioxidant effects of patuletin in neuronal cells (Khan et al., 2010) and in rats have also been reported (Abdel-Wahhab et al., 2005). ME is toxic to phytopathogenic fungi including *Botrytis cinera*, *Fusarium moniliforme*, and *Pythium ultimum* (Mares et al., 2004), while patuletin is also antibacterial, displaying toxicity towards *Micrococcus luteus*,* Corynebacterium*, *Staphylococcus*, and* Streptococcus* species (Faizi et al., 2008). In Pakistan, the powder of *T. patula *flower is also prescribed for the management of cancer patients (Azeemi, 1999). In our previous work, ME and the ethyl acetate (EA) fraction derived from ME demonstrated antiproliferative activity against cervical (HeLa), lung (NCI-H460), and skin (HT-144) cancer cells. In addition, patuletin was also found to be cytotoxic against the cervical (HeLa) cell line (Kashif et al., 2015). 

Genotoxic anticancer agents interact either directly with DNA (clastogenic, e.g., bleomycin) or with a mitotic apparatus, leading to unequal distribution of genetic material in daughter cells (aneugenic, e.g., vinblastine). These genetic damages appear as small spherical particles outside the nuclear membrane in the form of micronuclei formation and/or as chromosomal abnormalities in the cells. In many laboratories around the world, the *Allium *test is****widely used for genotoxic assessment due to its sensitivity, reliability, easy culturing, well-explored cell cycle kinetics (24 h), fast-dividing root tip cells, and correlation with mammalian test systems (Abdel Migid et al., 2007). The root tip cells of *Allium *species (*A. cepa* and *A. sativum*) have large-sized chromosomes (2n = 16) (Konuk et al., 2007), making them popular among researchers. Inhibition in root length accompanied by alterations in mitotic division and chromosomal structures of root tip cells are good indicators of cytotoxicity as well as genotoxicity (Hoshina and Marin-Morales, 2009). *A. cepa* was first used to determine the mutagenicity of inorganic salts (Levan, 1945). However, *A. sativum* has the advantage that each of its cloves can serve as a separate entity, thus reducing the amount of test samples required to perform experiments. 

The ME, EA, and pure compound patuletin were evaluated for their growth inhibitory activities to justify this plant’s traditional use in cancer. Growth inhibitory and cytotoxic effects were assessed by determining root length inhibition of *A.* *cepa* and/or *A. sativum* and the mitotic index of their root tip cells. In addition, their genotoxic actions as a mode of anticancer activity were assessed by analyzing the type of chromosomal abnormalities (CAs) and micronuclei formation in *Allium* root tip cells.

## 2. Materials and methods

Onion and garlic were purchased from a local market and other items were purchased from as follows: bleomycin hydrochloride (Bleocin, Nippon Kayaku Co. Ltd., Japan); vinblastine sulfate (Vinblas, Pharmedic Laboratories Pvt. Ltd., Pakistan); glass slides, glass cover slips (22 × 22 mm, Nr. 1., Citoglas, the Netherlands); acetic acid, orcein powder (Sigma, USA).

### 2.1. Collection and identification of T. patula 

The flowers of *T. patula* were identified by Dr. Rubina Dawar and collected and deposited in Herbarium of the Department of Botany, University of Karachi, Pakistan, as voucher specimen KUH GH No. 67280. The flowers were extracted and fractionated to obtain the pure compound patuletin as described earlier (Kashif et al., 2015).

### 2.2. HPLC fingerprinting of T. patula methanol extract, ethyl acetate fraction, and patuletin

High-performance liquid chromatography (HPLC) fingerprinting of ME and EA was conducted to detect the presence of patuletin. The purity of patuletin was also determined as described earlier (Li et al., 2010). Briefly, both samples and standards including patuletin, patulitrin, and methyl protocatechuate were prepared in the mobile phase. An HPLC-Prominence System Controller CBM-20A (Shimadzu) equipped with a degasser (DGU-20A5), quaternary pump (LC-20AT), autosampler (SIL-20A), and diode array detector (SPD-M20A) and software LC Solution were used for analysis. 

### 2.3. Solutions

The solutions of ME (25–1000 µg/mL), EA (1–100 µg/mL), and patuletin (5–100 µg/mL) were prepared in DMSO with final concentrations not exceeding 2%. Bleomycin (1–100 µg/mL) and vinblastine (1–50 µg/mL) were prepared in distilled water. 

### 2.4. Cytotoxic studies

The cytotoxic effects of ME, EA, and patuletin were determined by measuring root lengths as described earlier (Sehgal et al.,**2006) with slight modifications, i.e. the root inhibition was determined at 48 h, as described below.

#### 2.4.1. A. cepa

Conical and round onion bulbs weighing 50 ± 10 g with diameters of 17–19 cm were scratched until the root buds were visible without damaging the primordial root. The onions (n = 5) were grouped as: 1) control (d/w), 2) vehicle control (DMSO, 2%), and 3) treated with different concentrations of ME, bleomycin, and vinblastine. Only 5 healthy roots (2–3 cm) per onion were kept intact while the remaining roots were excised. After 48 h, the roots length were noted and photographed. 

#### 2.4.2. A. sativum

Garlic cloves (n = 30, ~2.0 g) were placed in glass vials for 3 days at 25 °C. On the 3rd day, 5 cloves/group were organized as follows: 1) control (d/w), 2) vehicle control (DMSO, 2%), and 3) treated with various concentrations of ME, EA, patuletin, bleomycin, and vinblastine. After 48 h, the roots length were recorded and photographed. 

### 2.5. Data handling for root length inhibition

The *Allium* root inhibition was determined by two methods, as described below. 

#### 2.5.1. Method-1 

*A. cepa* root length inhibition was determined by a commonly used method (Sehgal et al., 2006):

Root length inhibition (%) = Root length (in cm) [(Control – Treated) / Control] × 100.

#### 2.5.2. Method-2 

The mean root growth of *A. cepa* and *A. sativum* in the control and treated groups was determined as described earlier (Ali et al., 2017). Root growth was determined as follows: Root growth (cm) = Root length (cm) at (48 h – 0 h). Root growth inhibition (%) = Obtained root growth (cm) [(Control – Treated) / Control] × 100. The graph was plotted between various concentrations of test agents (x-axis) vs. percent root growth inhibitions (y-axis) and corresponding IC_50_ values were determined.

### 2.6. Microscopic studies of Allium species’ root tip cells 

The healthy *A. cepa* bulbs (n = 9) and *A. sativum* cloves (n = 9) were treated with respective IC_50_ values of ME, EA, patuletin, bleomycin, and vinblastine. Their roots were processed as described below.

#### 2.6.1. Harvesting, fixation, and hydrolysis of root tips

The root tips (~2–3 mm) from the control and respective treated groups were removed between 11:00 AM and 1:00 PM. These root tips (n = 27) were fixed (ethanol:acetic acid, 3:1, 24 h), hydrolyzed (1 N HCl solution, 5–15 min, to disengage the spatial arrangement between the cells), and rinsed with distilled water.

#### 2.6.2. Preparation and staining of slides 

The hydrolyzed root tips were dipped in aceto-orcein stain (2%, 30 min), placed onto a glass slide, smeared, and observed for mitotic stages under a microscope (Nikon TE-2000, Japan) at 400×. The photographs were further processed by Windows Photo Gallery and the relevant parameters were noted, as described below.

#### 2.6.3. Mitotic index 

In the control and treated groups, 459–749 cells/root tip were identified for the following stages. A) Interphase stage: the appearance of red, round/circular structure (nucleus) within the cell membrane. B) Mitotic stages: i) Prophase stage: thread-like chromosomes, ii) Metaphase stage: aligned chromosomes at the center, iii) Anaphase stage: chromatids move towards opposite poles, and iv) Telophase stage: two separate bundles of chromatids at opposite poles observed. The mitotic index was calculated as follows (Sehgal et al.,**2006):

Mitotic index (%) = (Cells in mitotic stages) / [Total cells (mitotic + interphase)] × 100.

#### 2.6.4. Chromosomal abnormalities (CAs)

The structural CAs in the control and treated groups were classified as i) bridging, ii) c-type chromosomes, iii) fragments, iv) lagging, v) misalignment, vi) multipolar, and vii) sticky chromosomes: 

CAs (%) = Structural changes in mitotic stages / Total cell count × 100.

#### 2.6.5. Micronuclei formation

The interphase stages of *A. cepa* and *A. sativum* root tip cells (at least ~14,000/group) were observed for the presence of micronuclei. These were recognized as tiny spherical nonrefractive nuclear particles visible as a dark red color, similar to that of the main nucleus in the interphase stage. 

### 2.7. Statistical analysis

The data for mitotic index, CAs, and micronuclei formation were transformed to minimize the variances in mean values by square root transformation method, i.e. (√(x + 1), where x is the mean value of the data. In all the experiments, the comparisons between controls and treated groups were made by one-way ANOVA and post hoc test (DMR, Dunnett’s multiple comparison test) using SPSS 12.0. Data with P < 0.05 were considered significant (Rebouças et al., 2013; Eke and Çelik, 2008).

## 3. Results

### 3.1. HPLC fingerprinting of ME, EA, and patuletin

**Figure 1:**
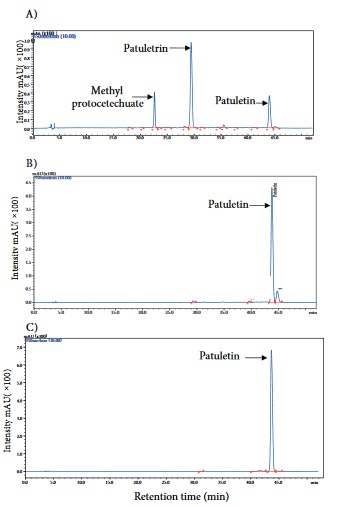
HPLC chromatograms of samples obtained from T. patula for the presence of patuletin. A) Methanol extract of T. patula flower, B) ethyl acetate fraction, and C) patuletin. Peaks are marked with arrows to signify the compound retention times. Detection wavelength: 350 nm. The unmarked minor peaks are unidentified and insignificant in the genotoxic results. Samples were eluted using mobile phases composed of aqueous phosphoric acid (0.1%, v/v) and acetonitrile. A gradient elution of acetonitrile, 5%–12% (0–12 min), 12%–16% (12–14 min), 16% B (14–21 min), 16%–20% (21–27 min), 20%–25% (27–37 min), was used. The reequilibration time of gradient elution was 15 min. The flow rate was 1.0 mL/min through a Hypersil Gold C8 column with diameter 250 × 46.6 mm and particle size 5 μm (Thermo Electron Corporation). The column temperature was maintained at 30 °C.

In Figure 1, ME displays three peaks demonstrating the presence of patuletin (~2.97%). However, in EA, it was ~36%. The purity of isolated patuletin was noted to be ~98.32%.

**Table 1:** Effect of methanol extract of T. patula flower on A. cepa root length.

Concentration (µg/mL)	Root length (cm) ± SEM		Time (h)		0	48
	100	2.41 ± 0.041	4.80 ± 0.057 (8 ± 0.47)
	200	2.47 ± 0.051,2	4.60 ± 0.066 (12 ± 0.5)
300	2.40 ± 0.051	4.35 ± 0.055 (17 ± 0.5)
400	2.47 ± 0.051,2	4.17 ± 0.064 (20 ± 0.36)
500	2.58 ± 0.052	3.98 ± 0.053 (24 ± 0.15)
600	2.49 ± 0.051,2	3.53 ± 0.062 (33 ± 0.58)
1000	2.43 ± 0.051,2	3.2 ± 0.051 (39 ± 1.04)
IC_50_	−	Not obtained
Control	2.43 ± 0.041,2	5.23 ± 0.078

Onion bulbs used (n = 9–12) and roots per onion (n = 5–7), total number of roots observed: control (distilled water) and methanol extract of T. patula (n = 44–60), each value represents mean ± SEM of 3 or 4 independent experiments. Values within parentheses represent the percentage root length inhibition as compared to the control. The inhibitory concentration that decreases the root length by 50% = IC_50_. In columns, dissimilar superscript numerals (1–8) represent significant differences (P < 0.05) while similar numerals represent nonsignificant root lengths compared to each other.

### 3.2. Root length and growth inhibition in A. cepa and A. sativum

**Figure 2:**
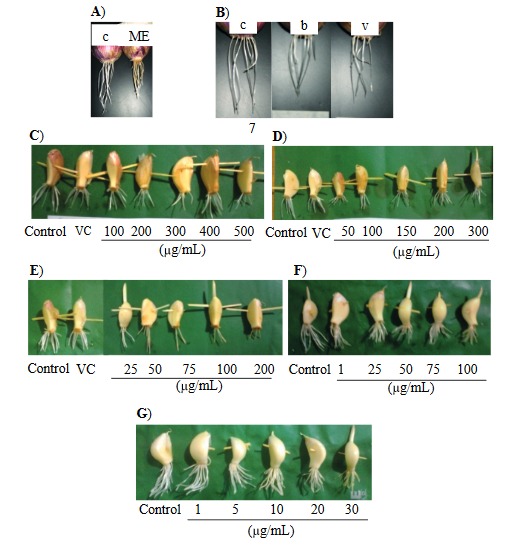
Root growth of A. cepa and A. sativum at 48 h. A. cepa root growth in A) (c) control and (ME) methanol extract of T. patula: 500 μg/mL; B) (c) control, (b) bleomycin (50 μg/mL), and (v) vinblastine (15 μg/mL). A. sativum root growth: C) methanol extract of T. patula flower, D) ethyl acetate fraction, E) patuletin, F) bleomycin, and G) vinblastine. In C, D, and E: (VC) vehicle control is 2% DMSO.

**Figure 3:**
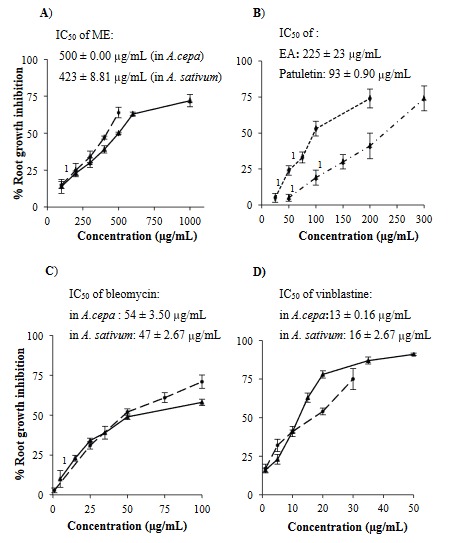
Effect on Allium root growth. A) Root growth inhibition of A. cepa ( ) and A. sativum ( ) in the presence of methanol extract of T. patula flower (ME). Control root growth (cm) in A. cepa = 2.80 ± 0.12, and in A. sativum = 1.32 ± 0.03. B) Root growth inhibition caused by ethyl acetate fraction (EA, ) and patuletin ( ) in A. sativum. Control root growth (cm) for ethyl acetate fraction = 1.62 ± 0.09, and patuletin = 1.59 ± 0.04. C) Root growth inhibition of A. cepa ( ) and A. sativum ( ) in the presence of bleomycin. Control root growth (cm) in A. cepa = 3.94 ± 0.11, and in A. sativum = 1.57 ± 0.09. D) Root growth inhibition of A. cepa ( ) and A. sativum ( ) in the presence of bleomycin. Control root growth (cm) in A. cepa = 3.72 ± 0.11, and in A. sativum = 1.65 ± 0.14. n = 52–60 root tips/concentration, percent root growth inhibition with superscript numeral (1) represents nonsignificant while without numerals indicates significant difference (P < 0.05) as compared to control root growth inhibition.

In *A. cepa* (n = 9) at 48 h, ME significantly reduced percent root inhibition (~8%–39%). However, the IC_50_ value could not be determined (Table 1). On the contrary, using root growth inhibition Method-2, the IC_50_ value was noted. In the case of EA-treated *A. sativum* roots, an IC_50_ value for root growth inhibition of ~225 µg/mL was observed, which was reduced by ~2.5 times in the presence of patuletin (Figures 2 and 3).

**Table 2:** Effect on mitotic stages, interphase, and mitotic index of A. cepa root tip cells.

Concentration (µg/mL)	Root length (cm) ± SEM		Time (h)		0	48
	100	2.41 ± 0.041	4.80 ± 0.057 (8 ± 0.47)
	200	2.47 ± 0.051,2	4.60 ± 0.066 (12 ± 0.5)
300	2.40 ± 0.051	4.35 ± 0.055 (17 ± 0.5)
400	2.47 ± 0.051,2	4.17 ± 0.064 (20 ± 0.36)
500	2.58 ± 0.052	3.98 ± 0.053 (24 ± 0.15)
600	2.49 ± 0.051,2	3.53 ± 0.062 (33 ± 0.58)
1000	2.43 ± 0.051,2	3.2 ± 0.051 (39 ± 1.04)
IC_50_	−	Not obtained
Control	2.43 ± 0.041,2	5.23 ± 0.078

Onion bulbs used (n = 9), roots per onion (n = 3), total number of root tips observed in control and with various concentrations of test samples (n = 27), each value represents mean ± SEM of 3 independent experiments. Values without parentheses represent the number of cells and those within parentheses are mean ± SEM of percentage of cells in various mitotic and interphase stages Mitotic Index (%) = Total cells in mitotic stages (a) / Total cells [mitotic stages (a) + interphase (b)] × 100. In columns, dissimilar superscript numerals (1–4) represent significant differences (P < 0.05) and similar numerals represent nonsignificant differences in mitotic stages, interphase, and mitotic index as compared to each other. Test agents used (IC_50_ values): T. patula flower methanol extract (500 μg/mL), bleomycin (50 μg/mL), and vinblastine (15 μg/mL).

**Table 3:** Effect on mitotic stages, interphase, and mitotic index of A. sativum root tip cells.

T. patula flower	Cells in mitotic stages								Prophase	Metaphase	Anaphase	Telophase	Total (a)	Interphase (b)	Total cells (a+b)	Mitotic index (%) a / (a+b)
	Methanol extract	3461,2,3(2.28 ± 0.15)	3131(2.06 ± 0.08)	1181,2(0.78 ± 0.09)	543(0.36 ± 0.02)	831	143502(94.53 ± 0.11)	15181	5.47 ± 0.112
Genotoxic drugs (positive control)								
Bleomycin (clastogenic)	1723(1.14 ± 0.10)	1682(1.12 ± 0.09)	942(0.62 ± 0.07)	892,3(0.59 ± 0.05)	523	145451(96.53 ± 0.14)	15068	3.48 ± 0.153
Vinblastine (aneugenic)	4091,2(2.50 ± 0.05)	2661(1.61 ± 0.16)	1172(0.72 ± 0.07)	1212(0.74 ± 0.11)	913	154952(94.43 ± 0.14)	16408	5.57 ± 0.142
Control	6461(3.46 ± 0.86)	3261(1.72 ± 0.18)	1871(1.00 ± 0.11)	2731(1.45 ± 0.12)	1432	173743(92.37 ± 1.00)	18806	7.63 ± 1.001

Garlic cloves used (n = 9), roots per garlic (n = 2), total number of root tips observed in control and with test samples (n = 18). The test agents used (IC_50_ values): T. patula flower methanol extract (500 μg/mL), ethyl acetate fraction (225 μg/mL), patuletin (100 μg/mL), bleomycin (50 μg/mL), and vinblastine (15 μg/mL). For more details see the legend of Table 2.

Similar to ME, bleomycin failed to display an IC_50_ value for *A. cepa* root length inhibition while vinblastine showed inhibition (data not shown). However, using Method-2, root growth inhibition in the presence of both bleomycin and vinblastine was noted (Figures 2 and 3).

### 3.3. Mitotic index

#### 3.3.1. A. cepa

In Table 2, it is evident that ME reduced cells in telophase stage by ~4.02 times as compared to the control. However, no changes in other mitotic stages were observed. In the presence of a clastogenic agent, i.e. bleomycin, all mitotic stages were significantly decreased. An aneugenic agent, i.e. vinblastine, caused decreases in only anaphase and telophase stages. The reduction in the mitotic index in the presence of ME was similar to vinblastine while it was 2 times less than that of bleomycin. 

#### 3.3.2. A. sativum

In Table 3,**ME demonstrates a significant decrease in prophase (~1.50×), anaphase (~1.31×), and telophase (~3.84×) stages with an increase in metaphase (~1.36×) as compared to the control. In the presence of EA and patuletin, a significant reduction in prophase (~2.36×), metaphase (~1.51×), anaphase (~1.45×), and telophase (~1.97×) was observed, which was also evident in bleomycin-treated cells. The mitotic index was significantly reduced by ~1.4 times in ME- and vinblastine-treated cells while it was ~2 times in the case of EA, patuletin, and bleomycin.

### 3.4. CAs

**Figure 4:**
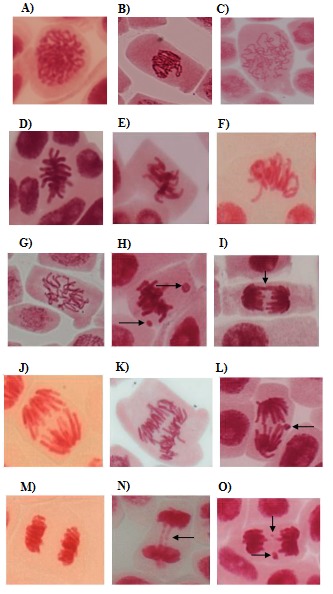
Representative chromosomal abnormalities in prophase, metaphase, anaphase, and telophase mitotic stages of Allium sativum root tip cells. Prophase: (A) normal, (B) sticky, and (C) c-type. Metaphase: (D) Normal, (E) misaligned, (F) sticky, (G) c-type, and (H) fragments. Anaphase: (I) Normal, (J) bridging, (K) multipolar, and (L) fragment. Telophase: (M) Normal, (N) bridging, and (O) fragments. In the presence of the methanol extract of T. patula flower (ME: 500 μg/mL) and vinblastine (15 μg/mL) predominant chromosomal abnormalities were those shown in B, C, E, F, G, J, K, and N, while in the presence of the ethyl acetate fraction (225 μg/mL), patuletin (100 μg/mL), and bleomycin (50 μg/mL) they were H, L, O. The arrows indicate corresponding chromosomal abnormalities. Magnification: 400×.

Various CAs induced by test agents at their respective IC_50_ values in *Allium* root tip cells are presented in Figure 4.

### 3.4.1. A. cepa

**Figure 5:**
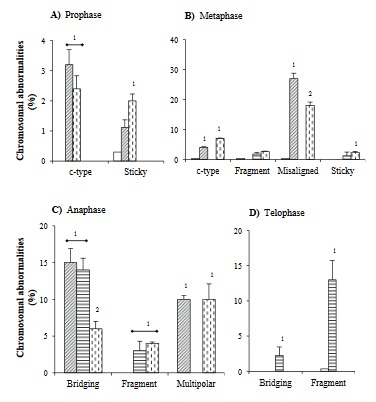
Chromosomal abnormalities in A. cepa root tip cells: ( ) control, ( ) methanol extract of T. patula flower-ME, ( ) bleomycin, and ( ) vinblastine. The percentages of cells with chromosomal abnormalities were calculated from total cells in respective mitotic stages (presented in Table 2). The bars without superscript numerals represent nonsignificant chromosomal abnormalities as compared to the control. Dissimilar superscript numerals (1–2) represent significant differences (P < 0.05) and similar numerals represent nonsignificant percentage values within particular chromosomal abnormalities. The test agents used (IC_50_ values): methanol extract of T. patula flower (ME: 500 μg/mL), bleomycin (50 μg/mL), and vinblastine (15 μg/mL).

In prophase stage, only ME and vinblastine significantly increased sticky and c-type CAs (Figure 5A). In metaphase stage, ME produced a significant increase in c-type CAs (~10×), which was doubled in the presence of vinblastine. Misaligned CAs with both ME and vinblastine (~90× and ~60×, respectively) were also present (Figure 5B). In anaphase cells, ME demonstrated bridging (~14×) of CAs with no fragments while both ME and vinblastine displayed multipolar (~10×) chromosomes. In contrast, bleomycin exhibited a significant rise in bridging (Figure 5C). In the telophase stage, only bleomycin induced chromosomal fragments (Figure 5D). 

It was also noted that ME- and vinblastine-treated cells had significantly increased cumulative CAs in prophase (~30×), metaphase (~80× and ~59×, respectively), and anaphase (~12× and ~9×, respectively) stages with no CAs in the telophase stage. However, in the case of bleomycin-treated root tip cells, CAs were significantly increased (~30×) only in the telophase stage. The potency order for the induction of CAs was ME > vinblastine > bleomycin.

### 3.4.2. A. sativum

**Figure 6:**
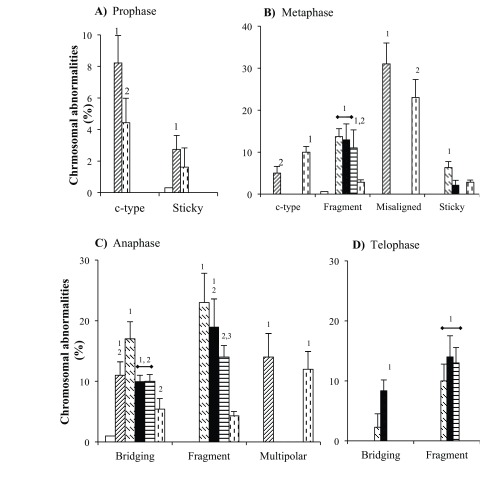
Chromosomal abnormalities in A. sativum root tip cells: ( ) ethyl acetate fraction, ( ) patuletin; for others refer to Figure 5. The percentages of cells with chromosomal abnormalities were calculated from total cells in respective mitotic stages (presented in Table 3). The bars without superscript numerals represent nonsignificant chromosomal abnormalities as compared to the control. Dissimilar superscript numerals (1–3) represent significant difference (P < 0.05) and similar numerals represent nonsignificant percentage values within particular chromosomal abnormalities. The test agents used (IC_50_ values): ethyl acetate fraction (225 μg/mL), patuletin (90 μg/mL); for others, refer to Figure 5.

In the prophase stage, only ME- and vinblastine-treated cells caused significant increases in c-type while only ME induced sticky chromosomes (Figure 6A). In the metaphase stage of ME- and vinblastine-treated cells, c-type and misaligned chromosomes were evident. On the other hand, EA, patuletin, and bleomycin displayed significantly higher rates of fragments (Figure 6B). In the anaphase stage of ME-treated cells, an increase in the bridged type of CA (~11×) was noted along with the induction of multipolar chromosomes (~12×), which was observed in vinblastine-treated cells. Conversely, EA, patuletin, and bleomycin caused an increase in fragmented chromosomes (~20%). In addition, EA also displayed the bridged type of CA (Figure 6C). In the telophase stage of EA-, patuletin-, and bleomycin-treated cells, only the fragment type (~10%) of CAs was noted (Figure 6D).

It was observed that ME-treated cells caused a significant production of CAs in the prophase stage (~30×), while in the presence of EA and patuletin, it was only in telophase phase (~3×). However, both groups presented higher CAs in metaphase (90× and 34×, respectively) and anaphase (~20× and ~39×, respectively) stages as compared to the control. In the case of bleomycin, a significant rise of CAs in metaphase (~20×), anaphase (~30×), and telophase (~2.22×) was evident. However, vinblastine produced significantly more CAs in prophase (~17×), metaphase (~80×), and anaphase (~20×). The potency order for the induction of CAs was ME > vinblastine > EA = patuletin = bleomycin.

### 3.5. Micronuclei formation

**Figure 7:**
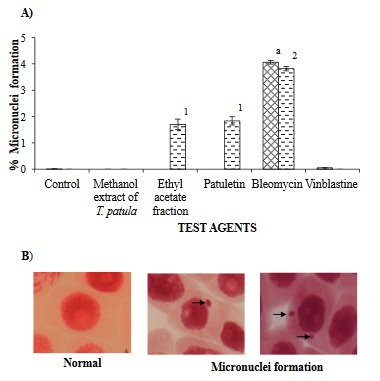
Micronuclei detection in A. cepa and A. sativum root tip cells. A) ( ) A. cepa, ( ) A. sativum. Total number of bulbs (n = 9), roots per onion (n = 3), total number of root tips observed in control (distilled water) and with various concentrations of test agents (n = 27). A. sativum: Total number of cloves (n = 9) and roots per garlic clove (n = 2), total number of root tips in control (distilled water) and with various concentrations of test agents (n = 18). Each value represents mean ± SEM of 3 independent experiments. The values without superscripts (letters or numerals) represent micronuclei formation similar to the control while those with superscript letters (a in A. cepa) or numerals (1–2 in A. sativum) are significantly (P < 0.05) different as compared to the control. In A. sativum, dissimilar superscript numerals (1–2) represent significant (P < 0.05) and similar numerals represent nonsignificant micronuclei formation when compared to each other. The treatments used (IC_50_ values): methanol extract of T. patula (500 μg/mL), ethyl acetate fraction (225 μg/mL), patuletin (100 μg/mL), bleomycin (50 μg/mL), and vinblastine (15 μg/mL). B) Representative photos of micronuclei formation in Allium root tip interphase cells of A. cepa.

Figure 7 displays that EA, patuletin (in *A. sativum*), and bleomycin (in both *A. cepa* and *A. sativum*) caused a significant rise in micronuclei formation.

## 4. Discussion

In the present study, the cytotoxic actions of ME, EA, and patuletin (flavonoid) were evaluated by root length inhibition and mitotic index in *A. cepa* and* A. sativum*. In addition, the types of structural CAs as well as micronuclei formations were also noted for the evaluation of aneugenic and/or clastogenic actions.

The *Allium* test resembles (~82%) mammalian test systems (Ray et al., 2013), making this test an efficient tool for the detection of toxicity of unknown chemicals. It utilizes mixed-function oxidases (Grant, 1982) for their metabolic activation. It can also determine genotoxins by identifying CAs and micronuclei formation. The IC_50_ values for root inhibition at 48 h is considered reliable for the assessment of the toxicity, genotoxicity (İlbaş et al., 2012), and cytogenetic actions (Konuk et al., 2007) of the compounds. However, IC_50_ values for *A. cepa *root inhibition in the presence of ME and bleomycin could not be determined using Method-1. The reason could be an unequal length of roots (ranging from 2 to 3 cm) at 0 h among the control as well as the treatment groups. Therefore, *A. cepa* root lengths of all the groups at 48 h were subtracted from their corresponding readings at 0 h to normalize the difference in root length and this was referred to as root growth (Ali et al., 2017). Using this approach, the IC_50_ value of root growth inhibition for ME in *A. cepa* and *A. sativum *was ~500 µg/mL and ~423 µg/mL, respectively, indicating that *A. sativum* is more sensitive than *A. cepa*, as has been observed earlier (Saxena et al., 2010). 

The *A. cepa* test was considered unsuitable for the assessment of EA or patuletin due to the requirement of large volumes of solutions (~300 mL). To cope with this problem, *A. cepa* seeds were tested for seed germination and growth of roots (data not shown), but they failed to fulfill the recommended criteria (Wang et al., 2001). Therefore, *A. sativum* cloves were used, which provided the advantage of being individual entities along with reduced requirements (~5-fold) of test solutions for the root inhibition studies. The IC_50_ values of EA and patuletin were ~2 and ~4 times lower than that of ME (~423 µg/mL), indicating that antiproliferative and cytotoxic action was increased in EA and patuletin as compared to ME. The potency order of root inhibition was patuletin > EA > ME. Earlier studies demonstrated root length inhibition in the presence of anticancer drugs. In the present study, the IC_50_ value obtained in the presence of tubulin inhibitor (vinblastine, IC_50_ ~13 µg/mL) displayed more potent effects in inhibiting root growth than a DNA-damaging agent (bleomycin, IC_50_ ~57 µg/mL). 

The inhibitory effect of toxic agents on root length is associated with a reduction in cell division (Ray et al., 2013), which can be determined by suppression of the mitotic index. The mitotic index provides information about the proportion of cells in various mitotic stages (M-phase) of the cell cycle, i.e. prophase, metaphase, anaphase, and telophase. In the present study, *T. patula* samples, vinblastine, and bleomycin reduced the mitotic index as compared to the control (~7.5%), which supported their cytotoxic action. The potency order of suppression of the mitotic index was EA = patuletin = bleomycin > ME = vinblastine. Furthermore, metaphase arrest in ME-treated *A. sativum* root tip cells was also evident. However, in ME- and vinblastine-treated *A. cepa* root tips cells, it was obtained when the metaphase:anaphase ratio was applied as described earlier (Jordan et al., 1992). Therefore, the mitotic data of tubulin disruptors (aneugenic) should be subjected to the metaphase:anaphase ratio to determine metaphase arrest. On the other hand, no metaphase arrest was observed in the *Allium* data for EA, patuletin, and bleomycin (clastogenic). In contrast, cells were arrested in the interphase stage, which might occur due to their direct genotoxic effects, decrease in cell numbers entering mitotic division, and the suppression of DNA synthesis by blocking the G1, G2, and/or S-phases (Türkoğlu, 2009), leading to alterations in the chromosomal structures. 

CA induction is a useful parameter of genotoxicity. In control *Allium* root tip cells, the presence of CAs (~0.80%) might be due to automutagenic effects (Ivanova et al., 2005) of genotype and environmental interactions. The ME, similar to vinblastine, induced higher rates of CAs (~17%–22%) than EA, patuletin, and bleomycin, indicating that all treatments possess genotoxic potential. The lower rates of CAs in EA, patuletin, and bleomycin are related to the reduced availability of cells in mitotic stages as compared to ME- and vinblastine-treated cells. Furthermore, ME- and vinblastine-induced CAs (c-mitosis, misaligned, and multipolar chromosomes) were mostly related to the interference with tubulin and spindle organization, indicating aneugenic action (Fernandes et al., 2009). The c-type chromosomes are usually formed as a result of microtubule and spindle abnormalities leading to scattered and chaotic chromosomes (Akyil et al., 2012). Misaligned chromosomes arise due to abnormal microtubules causing incomplete tubulin polymerization, which leads to random arrangement of chromosomes at the equatorial plate. Multipolar divisions are the result of problems in spindle fiber depolymerization that occur during polar shifting in metaphase and anaphase stages (Bhatta and Sakya, 2008). Additionally, in the presence of ME, lagging chromosomes were also indicated, which are also generated due to defects in spindle apparatus proteins (Liman et al., 2012). Conversely, EA, patuletin, and bleomycin displayed chromosomal fragments (~10%–20%), suggesting their clastogenic action. These are formed from chromosomal cuts, incompetent repair machinery, and interference in the structural organization of chromatin (Asthana et al., 2011). Some CAs were commonly noted in ME-, EA-, and patuletin-treated root tip cells including sticky (~3%–5%) and bridging (~10%–20%) CAs. Sticky chromosomes are clustered chromosomes associated with incomplete dissolution of the nucleoproteins that direct inappropriate chromosome fiber folding, inhibition of spindle formation, retardation of contraction and/or condensation of chromosomes, and improper depolymerization of DNA. It is an irreversible toxic effect resulting in cell death. Bridged chromosomes originate from sticky chromosomes due to improper chromosomal movement. They can also arise from fusion, breakage, and uneven distribution of chromatids during translocation (El-Ghamery et al., 2000). 

Micronuclei formation is another parameter for the determination of genotoxins. In the present study, control *A. sativum* cells in the interphase stage displayed no micronuclei while a few (~0.11%) were observed in *A. cepa*, similar to earlier reports on automutagenic effects (Ivanova et al., 2005). No micronuclei formation in the presence of ME (500 µg/mL) or vinblastine (15 µg/mL) might possibly be related to the absence of the fragment type of CAs, which leads to the formation of micronuclei (Hoshina and Marin-Morales, 2009). However, in *A. sativum* interphase cells, EA (225 µg/mL) and patuletin (90 µg/mL) induced micronuclei formation (~1.8%). This assures us that *T. patula* flower has genotoxic properties. Furthermore, its genotoxic potential should be explored in rodent bone marrow erythrocytes due to additional factors such as pharmacokinetics and repair systems. 

The HPLC fingerprinting of ME revealed that it contains ~3% patuletin (a flavonoidal molecule), which was increased to ~36% in EA. The IC_50_ value of root length inhibition for EA (225 µg/mL) involved patuletin of ~90 µg, which was similar to the IC_50_ of patuletin itself (purity of ~98%), suggesting that its cytotoxic and genotoxic activities are related to patuletin. This finding also corresponds to an earlier work in which the growth inhibitory effect of patuletin was ~6 and ~12 times higher than that of ME and EA, respectively, on cervical cancer (HeLa) cells (Kashif et al., 2015). Flavonoids possess antioxidant properties at concentrations between 5 and 25 µM, protecting cells from oxidative damage (Wätjen et al., 2005; Siddique and Afzal, 2009). However, flavonoids at higher concentrations produce genotoxic effects along with the following essential structural features: 1) double bond between carbons 2 and 3 of the C-ring, 2) OH- group at carbon 3 of the C-ring, 3) OH- group at carbon 7 of the A-ring, and 4) catechol or pyrogallol on the B-ring (Silva et al., 2000). The clastogenic action of flavonoids at higher concentrations could be induced by intercalation of DNA, topoisomerase II inhibition, and generation of free radicals (Snyder and Gillies, 2002). 

In conclusion, ME, EA, and patuletin possess cytotoxic and genotoxic actions. In both *Allium* species, ME acts as an aneugen (comparatively higher mitotic index with metaphase arrest, formation of c-type and misaligned chromosomes, and no detectable micronuclei formation), while EA and patuletin are clastogenic (low mitotic index, chromosomal fragments, induction of micronuclei) in action. However, further preclinical and clinical studies are required to determine the clinical efficacy of *T. patula *flower against human cancers.

## Acknowledgments

We would like to thank Dr Nadia Khan, Department of Genetics, University of Karachi, for her help with the statistical analysis. We are also thankful to the Higher Education Commission of Pakistan for the scholarship to Samina Bano (Indigeneous 5000 Fellowship Program Batch V). Our utmost appreciation goes to Dr. Rubina Dawar (taxonomist from Department of Botany, University of Karachi) for the identification of the plant.
